# An optimal B-spline approach for vectorizing raster image outlines

**DOI:** 10.1371/journal.pone.0307530

**Published:** 2024-08-26

**Authors:** Samreen Abbas, Malik Zawwar Hussain, Qurat ul-Ain

**Affiliations:** 1 Department of Mathematics, GC Women University, Sialkot, Pakistan; 2 Department of Mathematics, University of the Punjab, Lahore, Pakistan; 3 Department of Mathematics, University of Central Punjab, Lahore, Pakistan; Public Library of Science, UNITED KINGDOM OF GREAT BRITAIN AND NORTHERN IRELAND

## Abstract

Capturing outlines is crucial in vectorization of the digital images. An effective and automatic algorithm is presented for outline vectorizing of planar objects within the digital images using trigonometric B-spline. A soft computing optimization technique Genetic Algorithm (GA) is employed to determine the suitable measures of parameter in the description of proposed B-spline. The anticipated scheme is executed on a few raster (bitmap), digital images to validate the robustness of the algorithm. The procedure of vectorizing outlines encompasses a series of stages like boundary detection, corner recognition, break point identification and curve fitting using the proposed B-spline.

## 1. Introduction

The majority of computer images are typically in raster format. These images are prevalent due to the fact that nearly all scanners and digital cameras generate raster images. These raster images consist of minuscule dots referred to as pixels, with each pixel representing the color of the image at a specific location. Typically, these pixels are organized in a regular rectangular grid. On the other hand, there exists an alternative image format known as vector format. In contrast to raster images, which are composed of pixels, vector images consist of smoother elements such as lines, polygons, and curves. Furthermore, vector images often occupy less storage space, with the required space being determined by the complexity of the image rather than its size and resolution. Due to the advantages associated with vector images, the ability to convert a raster image into a vector image is highly valuable. This process of conversion is cited to as vectorization. Instead of concentrating on scaling, sharing, and rotational aspects involved in vectorization for these images, the motivation for the proposed work is focused specifically on image outline vectorization.

One of the fundamental features of capturing and vectorizing image outlines entails the modeling and designing of appropriate curve fitting schemes. This stage of dealing with image outlines makes essential significances when addressing real-world applications involving in cartooning, logo designing, font designing, embroidery and textile designing among others. Whereas other stages of the procedure consist of a number of computational and mathematical phases, all of which are usually intended to approximate a curve that closely fits the set of data points. Hence, the long-term objective of this effort is to generate vectorizing outline for planar objects in the image that are both approximately reliable and computationally effective. There are many benefits to using curves to express planar things. While substantial progress has been made in this field through previous research efforts [1–12], there remains a desire to delve further into more advanced and interactive strategies.

In this work, an optimal approach for image vectorizing outline is presented, categorically designed to handle the data gathered from the raster (bitmap) image boundaries for planner objects. To achieve optimal curve fitting for image outline, the suggested method uses a free-form trigonometric B-Spline representation and the capabilities of soft computing, specifically the Genetic Algorithm (GA). In this framework, a quadratic trigonometric B-spline has been used, which is defined by a single shape parameter that is introduced in its description. In contrast, GA seeks to identify parameter values that minimize the discrepancy between the fitted spline curve and the observed image boundary.

Trigonometric B-spline as compare to traditional B-splines are more versatile in dealing with curve fitting complex geometrical challenges. In contrast to traditional B-spline, they utilized trigonometric basis functions involving in sine and cosine functions aeeeeeeeeelong a with series of control points. By hiring these trigonometric B-spline curves in vectorization process, the algorithm acquires the ability to accurately capture and flexible representation of curve to complex geometrical shapes and counters presented in raster images. This flexibility enables seamless handling of curves, resulting in better vectorization outcomes particularly when the form displays periodic or wave-like features.

Genetic Algorithms (GAs) have proven to be useful and effective in variety of sectors that deals with optimization challenges. The foundation of these algorithmic techniques is the idea of natural selection and evaluation. By applying GAs to proposed image vectorization procedure, they offer powerful approach to automate and optimized the values of parameters of trigonometric B-splines to get the best suited incoming raster image data. The optimization procedure iteratively modified the control points, basis function and other parameters of trigonometric B-spline curves and enable the generation of high quality vectorized presentation with minimal manual intervention, that helps to reduce the inaccuracy between the newly generated vectorized outline with original one. Cumulatively, use of trigonometric B-spline along with GAs into the proposed vectorization scheme, enhanced the capabilities to capture complex geometric shapes and optimizes the vectorized outline.

The paper is structured as: Section 2 and Section 3 covers the construction and properties of proposed free form trigonometric B-Spline like representation. Section 4 discusses the genetic algorithm, while Section 5 describes the overall proposed approach, including boundary extraction, corner identification, problem matching and curve fitting. Section 6 demonstrates all the pictorial results from iterations when applied to the sample images whereas the comparative and subsequent studies are conducted in Section 7. Finally, the concluding remarks are made in Sections 8.

## 2. Curve fitting with Quadratic Trigonometric B-spline (QTB-Spline)

A space of QTB-Spline function with single control parameter is developed by using a constructive approach described in [13]. A quadratic trigonometric spline with parameter *ω*_*i*_ is built up in Section 2.1 to formulate the above said QTB-Spline followed by the construction of free form trigonometric basis and B-spline like curve modelling in Section 2.2 and Section 2.3 respectively.

### 2.1 Quadratic Trigonometric Spline (QTS)

A Quadratic Trigonometric Spline (QTS) space Ω(Δ), where Δ = ($a^{*}=\zeta_{0}<\zeta_{1}<\zeta_{2}<\cdots <\zeta_{n}=b^{*}$) for *n* ∈ *N*, is put in a space of *C*^1^ trigonometric piecewise polynomials of degree ≤ 2. The QTS space is spanned by the QT basis functions, $\;R_{k,i}(\Theta ,\omega_{i});\;k=0,1,2,3$ with control parameter *ω*_*i*_∈[−1,1], defined in each subinterval [*ζ*_*i*_, *ζ*_*i*+1_] as:

$$\begin{array}{l} R_{0,i}(\Theta ,\omega_{i})=(1-\sin\Theta )(1+(1-\omega_{i})\sin\Theta )\\ R_{1,i}(\Theta ,\omega_{i})=\omega_{i}\sin\Theta (1-\sin\Theta )\\ R_{2,i}(\Theta ,\omega_{i})=\omega_{i}\cos\Theta (1-\cos\Theta )\\ R_{3,i}(\Theta ,\omega_{i})=(1-\cos\Theta )(1+(1-\omega_{i})\cos\Theta ) \end{array}\}$$ (1)

with $\Theta =\frac{\pi }{2}\delta^{*}$ and $\delta^{*}=(\frac{\zeta -\zeta_{i}}{\delta_{i}})$.

**Fig 1:**
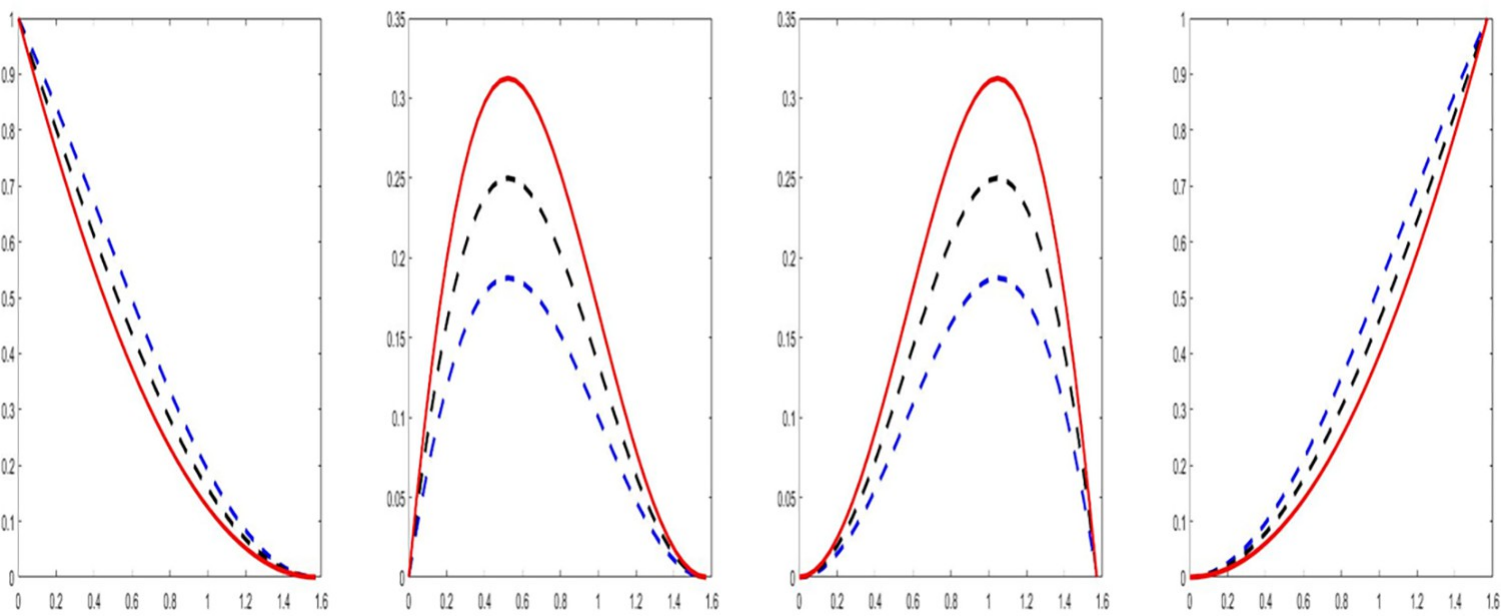
The quadratic trigonometric basis functions for *ω*_*i*_ = 0.5; 0.8; 1.

It can easily be noticed from Eq (1) that the QT basis functions have the similarity to the basis functions of Bernstein B*é*zier which are demonstrated in Fig 1.

### Proposition1

Th geometric features of the basis functions defined in (1) are as follows:

$$•\;Positivity:\;R_{k,i}(\Theta ,\omega_{i})\geq 0;\;k=0,1,2,3,i=1,2,\ldots ,n-1.$$ (2)

$$•\;Partition\;of\;Unity:\;\sum_{k=0}^{3}R_{k,i}(\Theta ,\omega_{i})=1;\;i=1,2,\ldots ,n-1.$$ (3)

$$•\;Symmetry:\;R_{k,i}(\Theta ,\omega_{i})=R_{3-k,i}(\Theta ,\omega_{i});\;k=0,1,2,3,\;i=1,2,\ldots ,n-1.$$ (4)

**Proof.** The above-mentioned characteristics arise directly from the formulation and evolution of the basis function.

### 2.2 Construction of free form trigonometric basis

On the way to The approach of this section is to furnish the trigonometric B-spline free form basis of QTS defined in Section 2.1. For the local function *η*_*k*_(*ζ*); *k* = −1,…,*n*+3 with suggested spline, is defined to construct trigonometric free form basis. Let us take, additional knots $\zeta_{-3}<\zeta_{-2}<\zeta_{-1}$ and $\zeta_{n+1}<\zeta_{n+2}<\zeta_{n+3}$, both sides of the interval [*ζ*_0_, *ζ*_*n*_]. The local quadratic function *η*_*k*_(*ζ*); *k* = −1,…,*n*+3 is defined as:

$$\eta_{k}(\zeta )=\left\{ \begin{array}{c} 0\;for\;\zeta <\zeta_{k-2}\\ 1\;for\;\zeta \geq \zeta_{k+1} \end{array}\right.$$ (5)

and

$$\eta_{k}(\zeta )=R_{0,i}(\Theta ,\omega_{i})\eta_{k}(\zeta_{i})+R_{1,i}(\Theta ,\omega_{i})\left(\eta_{k}(\zeta_{i})+\frac{2\delta_{i}}{\pi \omega_{i}}\eta_{k}^{\left(1\right)}(\zeta_{i})\right)$$

$$+R_{2,i}(\Theta ,\omega_{i})\left(\eta_{k}(\zeta_{i+1})-\frac{2\delta_{i}}{\pi \omega_{i}}\eta_{k}^{\left(1\right)}(\zeta_{i+1})\right)+R_{3,i}(\Theta ,\omega_{i})\eta_{k}(\zeta_{i+1})$$ (6)

for the intervals $[\zeta_{i},\zeta_{i+1}),i=k-2,k-1,k$ where $R_{k,i}(\Theta ,\omega_{i});\;k=0,1,2,3$ are defined in the same way as in Eq (6). As per necessity that the function *η*_*k*_ is second order continuous at $\zeta_{k-2},\zeta_{k-1},\zeta_{k\;}\;\mathrm{and}\;\zeta_{k+1}$, the function *η*_*k*_(*ζ*) must satisfied the following:

ηk(ζk−2)=0,ηk(1)(ζk−2)=0ηk(ζk−1)=ξk−1,ηk(1)(ζk−1)=ξk−1^ηk(ζk)=1−ϵk,ηk(1)(ζk)=ϵk^ηk(ζk+1)=1,ηk(1)(ζk+1)=0} (7)

where,

ξk−1=δk−2πξk−1^,ξk−1^=δk−2vkuk

ϵk=δkπϵk^,ϵk^=δkvk−1uk

$$v_{k}=[\frac{\delta_{k}}{\pi }+\frac{\delta_{k-1}}{\pi }]\;,\;u_{k}=(\delta_{k-2}+\delta_{k-1}+\delta_{k})v_{k}v_{k-1}$$

Next, the difference of the function *η*_*k*_ will be took, for the final expression of free form trigonometric free form basis, as:

$$M_{k}(\zeta )=\eta_{k}(\zeta )-\eta_{k+1}(\zeta ),\;k=-1,\ldots ,n+1$$ (8)

A definite form of the trigonometric basis function *M*_*k*_(*ζ*) on any interval [*ζ*_*i*_, *ζ*_*i*+1_) is composed by (6)–(8) as:

$$M_{k}(\zeta )=R_{0,i}(\Theta ,\omega_{i})M_{k}(\zeta_{i})+R_{1,i}(\Theta ,\omega_{i})\left(M_{k}(\zeta_{i})+\frac{2\delta_{i}}{\pi \omega_{i}}M_{k}^{\left(1\right)}(\zeta_{i})\right)+$$

$$R_{2,i}(\Theta ,\omega_{i})\left(M_{k}(\zeta_{i+1})-\frac{2\delta_{i}}{\pi \omega_{i}}M_{k}^{\left(1\right)}(\zeta_{i+1})\right)+R_{3,i}(\Theta ,\omega_{i})M_{k}(\zeta_{i+1})$$ (9)

where

$$M_{k}(\zeta_{i})=M_{k}^{\prime }(\zeta_{i})=0,\;for\;i\neq k-1,k,k+1$$

Mk(ζk−1)=ξk−1,Mk(1)(ζk−1)=ξk−1^,Mk(ζk)=1−ϵk−ξk,

Mk(1)(ζk)=ϵk^−ξk^,Mk(ζk+1)=ϵk+1,Mk(1)(ζk+1)=−ϵk+1^,

#### Proposition 2

The geometric feature of trigonometric free form basis functions (9) are as follows:

$$•\;Local\;Support:\;M_{k}(\zeta )=0,\;for\;\zeta \notin (\zeta_{k-2},\zeta_{k+2})$$ (10)

$$•\;Partition\;of\;unity\;property:\sum_{k=-1}^{n+1}M_{k}(\zeta )=1,\;for\zeta \in [\zeta_{0},\zeta_{n}]$$ (11)

$$•\;Property\;of\;positivity:\;M_{k}(\zeta_{k})>0,\;for\;all\;\zeta .$$ (12)

**Proof:** The above-mentioned characteristics arise directly, from the formulation and growth of the basis function.

### 2.3 B-Spline like curve modelling

To compute the required Quadratic Trigonometric B-spline (QTB-Spline) freeform cure representation, let us defined P*(*ζ*) for *ζ*∈[*ζ*_0_, *ζ*_*n*_] as:

$$P^{*}(\zeta )=\sum_{k=-1}^{n+1}M_{k}(\zeta )T_{k}^{*}\;,\;\forall \zeta \in [\zeta_{0},\zeta_{n}]$$ (13)

With control points $T_{k}^{*}$, whose value can be found with help of set of given spatial data points. Furthermore, Eq (13) can be rewritten as:

$$P^{*}(\zeta )=\sum_{k=i-1}^{i+2}M_{k}(\zeta )T_{k}^{*},\forall \zeta \in [\zeta_{i},\zeta_{i+1}),i=0,1,\ldots ,n-1$$ (14)

From Eq (9) and Eq (14);

$$P^{*}(\zeta )=R_{0,i}(\Theta ,\omega_{i})L_{k}+R_{1,i}(\Theta ,\omega_{i})U_{k}+R_{2,i}(\Theta ,\omega_{i})V_{k}+R_{3,i}(\Theta ,\omega_{i})L_{k+1}$$ (15)

where

$$L_{k}=\epsilon_{k}T_{k-1}^{*}+(1-\epsilon_{k}-\xi_{k})T_{k}^{*}+\xi_{k}T_{k+1}^{*}$$

$$U_{k}=\alpha_{k}\epsilon_{k}T_{k-1}^{*}+(1-\alpha_{k}\epsilon_{k}-\beta_{k}\xi_{k})T_{k}^{*}+\beta_{k}\xi_{k}T_{k+1}^{*}$$

$$V_{k}=\beta_{k}\epsilon_{k+1}T_{k}^{*}+(1-\beta_{k}\epsilon_{k+1}-\alpha_{k}\xi_{k+1})T_{k+1}^{*}+\alpha_{k}\xi_{k+1}T_{k+2}^{*}$$

with $\alpha_{k}=(1-2/\omega_{k})$ and $\beta_{k}=(1+2/\omega_{k})$,

Let ${P^{*}}_{k}={\left[L_{k\;}\;\begin{array}{ccc} U_{k} & V_{k} & L_{k+1} \end{array}\right]}^{t}$ and $Z_{k}={\left[{T^{*}}_{k-1\;}\;\begin{array}{ccc} {T^{*}}_{k} & {T^{*}}_{k+1} & {T^{*}}_{k+2} \end{array}\right]}^{t}$ and


$${Q^{*}}_{k}=\left[\begin{array}{c} {R_{0}\epsilon }_{k+}R_{1}\alpha_{k}\epsilon_{k}\\ R_{0}(1-\epsilon_{k}-\xi_{k})+R_{1}(1-\alpha_{k}\epsilon_{k}-\beta_{k}\xi_{k})+R_{2}\beta_{k}\epsilon_{k+1}+{R_{3}\epsilon }_{k+1}\\ R_{0}\xi_{k}+{R_{1}\beta }_{k}\xi_{k}+R_{2}(1-\beta_{k}\epsilon_{k+1}-\alpha_{k}\xi_{k+1})+R_{3}(1-\epsilon_{k+1}-\xi_{k+1})\\ {R_{2}\alpha }_{k}\xi_{k+1}+{R_{3}\xi }_{k+1} \end{array}\right]$$

Eq (9) can be reconstructed in vector form as:

$${P^{*}}_{k}={Q^{*}}_{k}∙Z_{k}.$$ (16)

Now, the transformed Bernstein Be´zier in Eq (16) can easily be used for computational purpose. It’s important to highlight that the challenge of QTB-Spline interpolation can be addressed through the following equation, where the diagonal-dominating tridiagonal matrix *M*_*j*_(*ζ*_*j*_) is involved:

$$\sum_{k=-1}^{n+1}M_{k}(\zeta_{k})T_{k}^{*}=L_{k\;},\;\forall k$$

**Fig 2:**
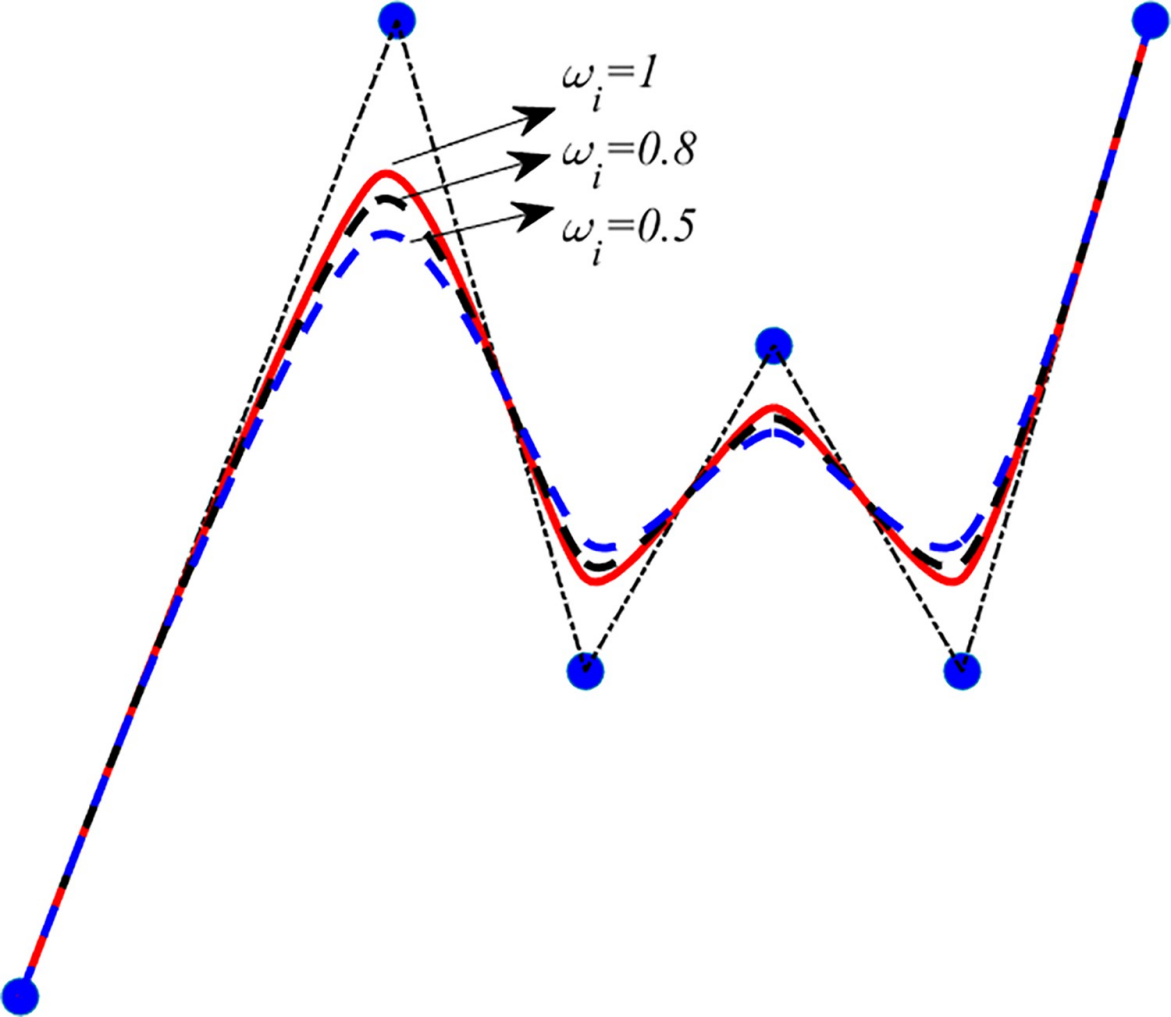
The QTB-spline curves with different shape parameters.

This equation establishes the existence of a unique QTB-Spline as an alternative to B-splines. Fig 2 provides some examples about the adjustment effect of shape parameters *ω*_*i*_ on the QTB-spline curve given in (16), thus the QTB-spline curve shows better approximation ability than classical quadratic Be´zier curves.

## 3. Geometric properties of QTB-Spline

### Proposition 3

[Convex Hull Property (CHP)]: The QTB-Spline curve completely lies inside the convex hull produced (CHP) by the control polygon for {*L*_*k*_, *U*_*k*_, *V*_*k*_, *L*_*k*+1_}.

#### Proof

Given that from (11) and (12),

$$\sum_{k=-1}^{n+1}M_{k}(\zeta )=1,\;for\;\zeta \in [\zeta_{0},\zeta_{n}]\;\mathrm{and}\;M_{k}(\zeta )>0,\;\mathrm{for}\;\mathrm{all}\;\zeta .$$

**Fig 3:**
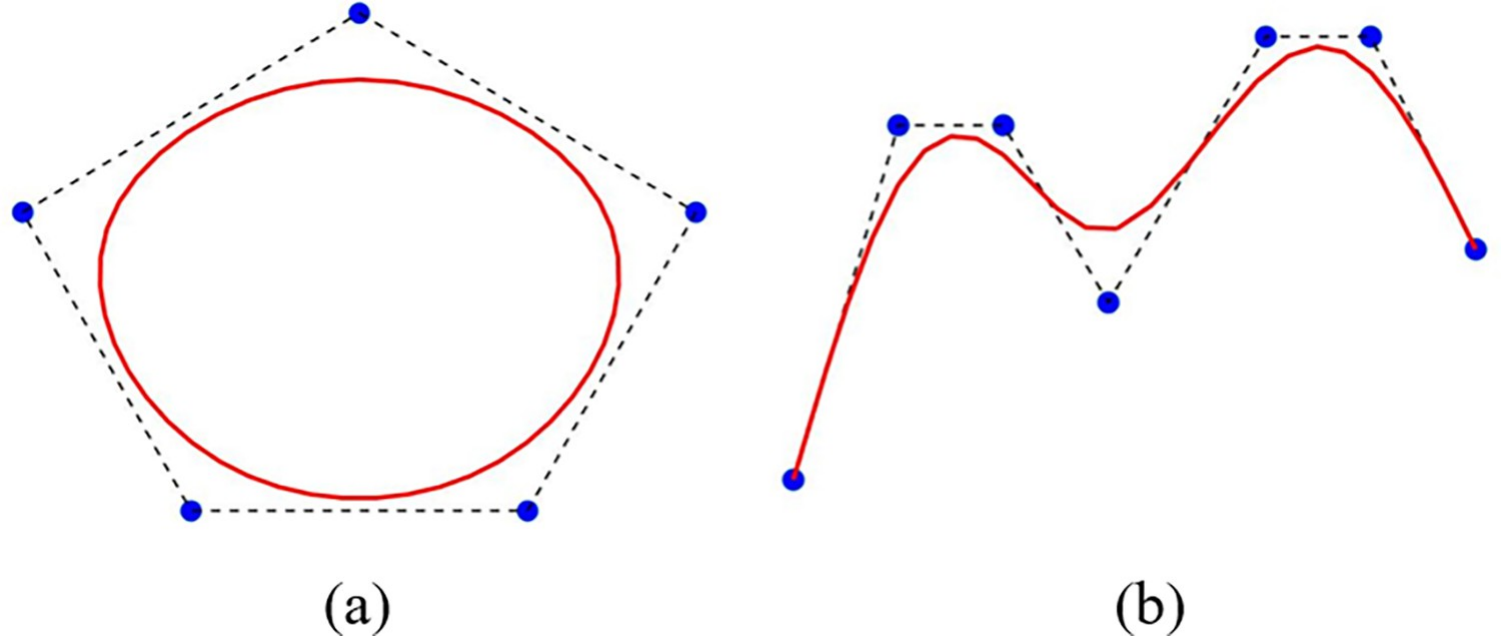
Graphical view of CHP.

Consequently, the QTB-Spline curve is entirely held within the convex hull outlined by its control polygon, which is formed by {*L*_*k*_, *U*_*k*_, *V*_*k*_, *L*_*k*+1_} as depicted in Fig 3(A) and 3(B).

#### Proposition 4

[Variation Diminishing Property (VDP)]: Consider $P(\zeta )=\sum_{k=0}^{3}M_{k}(\zeta )T_{k}^{*}$, where *ζ* belongs to the interval [*ζ*_0_, *ζ*_*n*_]. For the QTB-Spline curve, almost any N-1 ultra dimensional plane intersects the curve only by following the control polygon set by the control points {*L*_*k*_, *U*_*k*_, *V*_*k*_, *L*_*k*+1_}.

#### Proof

Derived from (15) it becomes evident that both *ϵ*_*k*_, *ξ*_*k*_≥0. Furthermore, *U*_*k*_ and *V*_*k*_ reside on the line that connects *L*_*k*_ and *L*_*k*+1_. As, $\mu_{k}=\frac{h_{k}}{h_{k}-h_{k-1}}$ and fall within the range 0 < *μ*_*k*_ < 1, the following relationship can be expressed as:

$$L_{k}=(1-\mu_{k})U_{k-1}+\mu_{k}V_{k}$$

**Fig 4:**
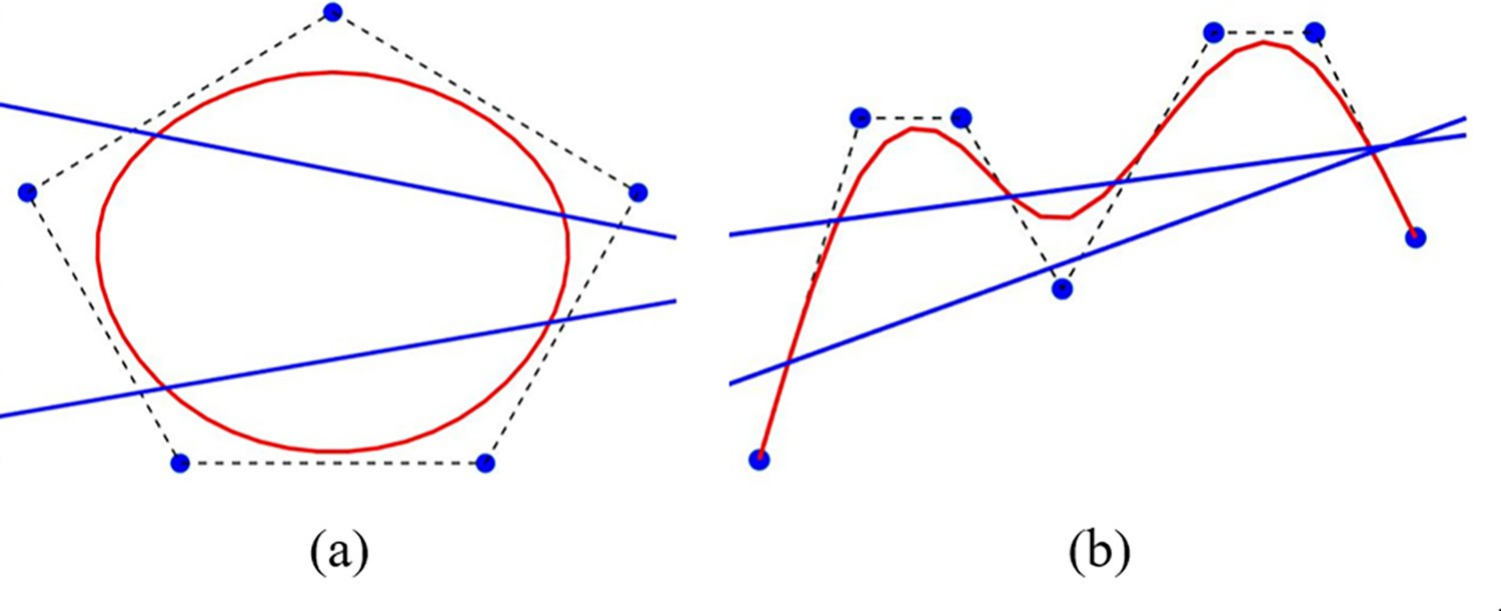
Graphical view of VDP.

Consequently, the control points of QTB-Spline control polygon result in similar way that the polygon of piecewise Bernstein Be´zier do. The QTB-Spline adheres to the variation diminishing property, as this property is satisfied by the the piecewise Bernstein Be´zier curve. Fig 4(A) and 4(B) visually exemplify the VDP for the QTB-Spline.

## 4. Genetic Algorithm (GA)

**Fig 5:**
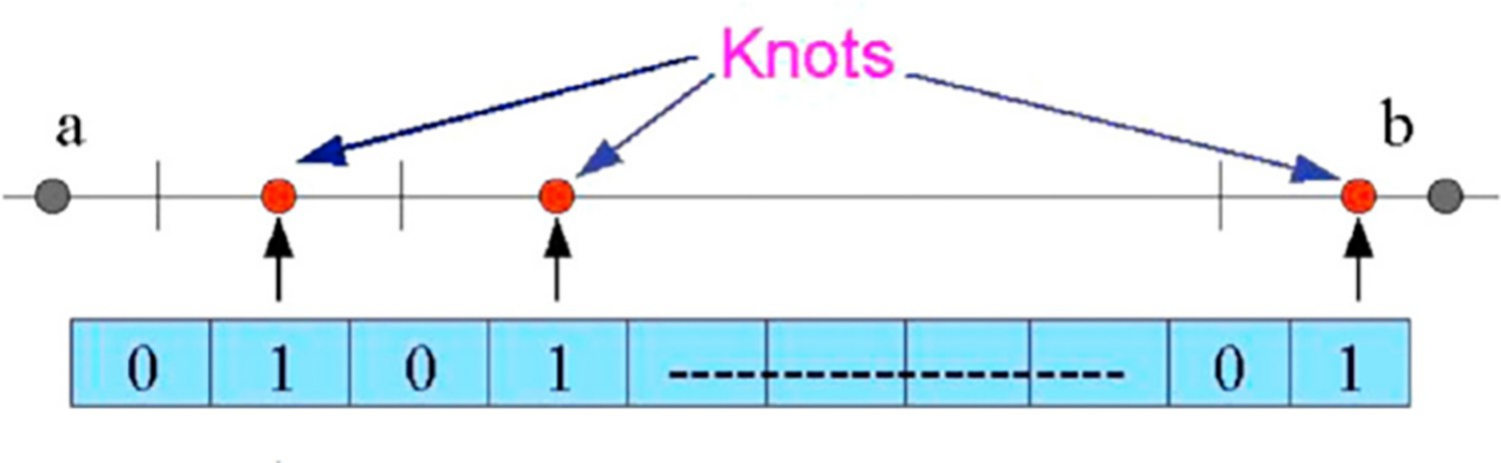
An individual chromosome within the genetic framework.

GA [14] is a search technique inspired by the natural procedure of evolution. It is employed to generate solutions for optimization problems. In this method, each potential solution within the search space is represented as a bitstring, referred to as a chromosome, with its individual bits being the genes, as shown in Fig 5. GA commences with a set of potential solutions known as a population. It operates with genetic optimization operators: mutation, selection operator and crossover, in conjunction with a fitness function. The primary aim of the crossover and mutation operators is to create new chromosomes from existing ones, inheriting beneficial traits from their parent chromosomes. The fitness function assesses the quality of each within the population, with higher fitness values increasing the likelihood of selection.

Following the selection of the fittest chromosomes from the mutation, population and crossover, operations are enforced to enhance the fitness values. Before initiating the search, GA randomly selects a collection of chromosomes from the search space, where in our specific problem, the chromosomes represent the shape parameters. Subsequently, optimal operators are executed to generate new offspring, with the anticipation that each new generation will exhibit improved fitness compared to the previous one. Only chosen chromosomes are allowed to reproduce. The iterative application of this algorithm gradually produces the best chromosome, and the process continues until a predefined stopping condition is satisfied, ultimately yielding a solution to the problem. Table 1 provides a list of the GA parameters employed in the proposed scheme.

**Table 1:** Parameters of GA.

Parameter	Values
Population Size	30
Mutation Rate	0.001
Selection Rate	0.5
Genome Length	10
Threshold	3

## 5. Implementation strategy

**Fig 6:**
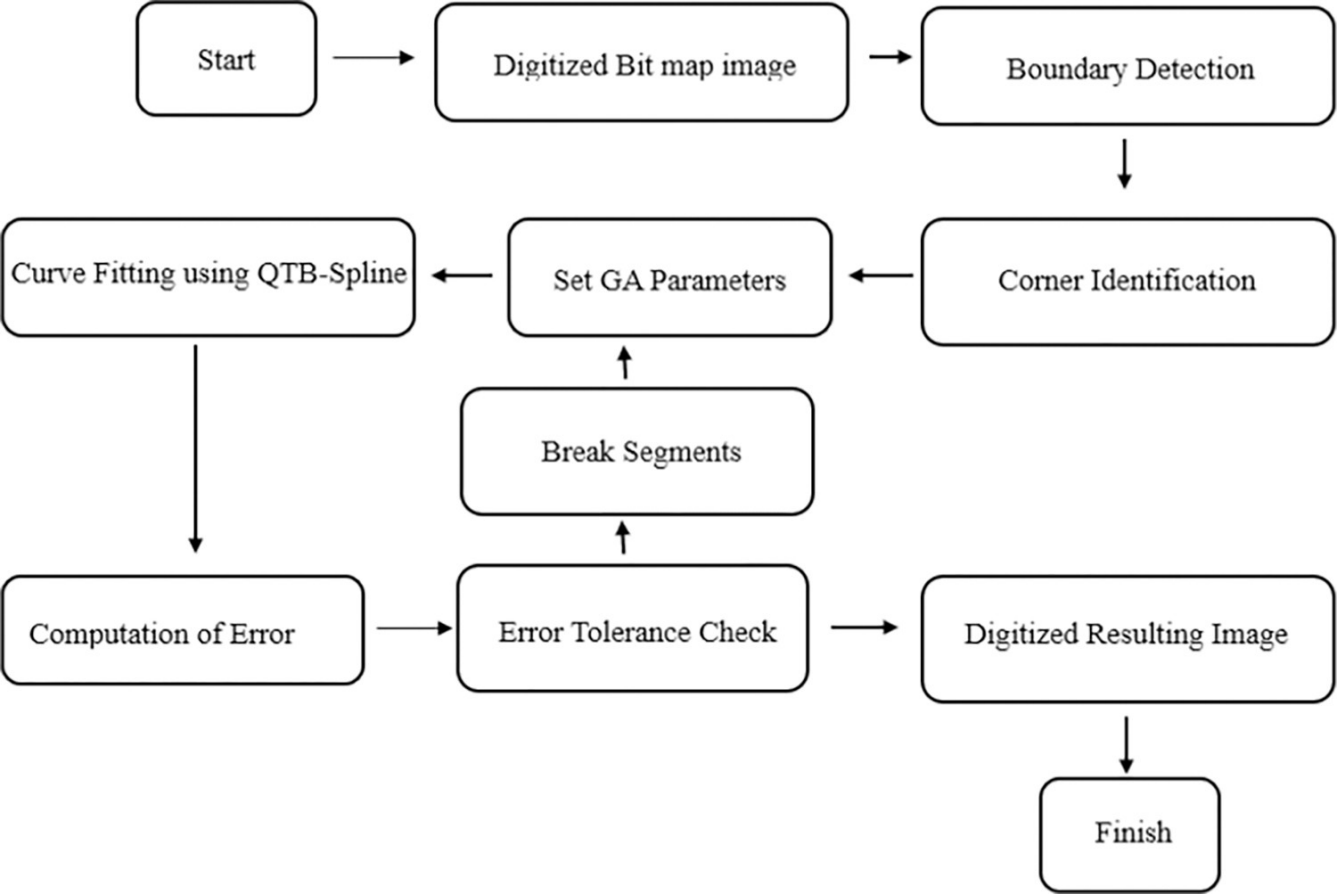
Flow chart of proposed approach.

The comprehensive plan for addressing the problem at hand is elaborated upon in this discussion. The entire algorithm is visually depicted in Fig 6, showcasing the flowchart of the process.

The goal is to establish a curve that optimally matches the data generated by the boundaries of planar objects in images. The proposed approach encompasses several stages, including boundary detection, identification of corner points, curve fitting through the utilization of Genetic Algorithms and the trigonometric B-spline representation (16).

### 5.1 Boundary detection

The process of obtaining the graphical representation of planar objects is initiated by detecting their boundaries. The prevalent method for extracting these boundaries is detailed in [15]. This technique operates by taking the image of the object as input and subsequently deriving chain codes that correspond to the direction of the edges for each contour. These chain-coded curves serve as the output, effectively defining the object’s boundary within the image. Extracted boundaries of the bitmap images given in Figs 9(A), 10(A) and 11(A) are in Figs 9(B), 10(B) and 11(B), respectively.

### 5.2 Identification of corner points

**Fig 7:**
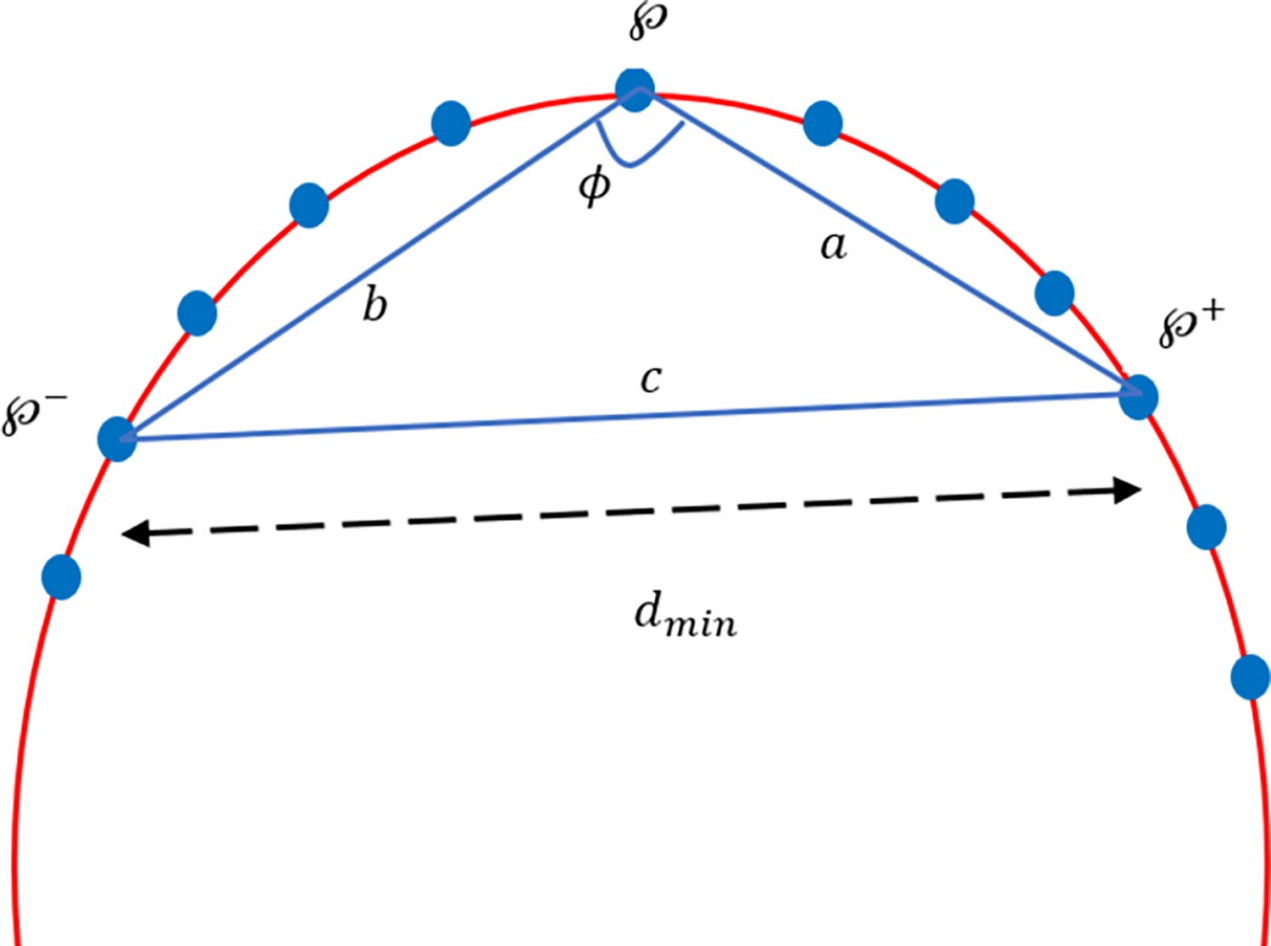
Corner point detection demonstration—Phase 1.

Corners play a valuable role as they offer a preliminary insight into the configuration of an image. The approach used for corner identification in this paper is expounded in [16], wherein corner intensity is linked with each point along the boundary. The procedure comprises two distinct phases. In the initial phase, it identifies the corners from the entire dataset, while in the second phase, certain criteria are applied to eliminate additional points, retaining only possible corners. The details of these phases provided in [16] are as:

Phase 1: In this phase a variable triangle (℘^−^,℘,℘^+^) is recruited on each point of the curve as shown in Fig 7. A point ℘_*i*_ is the corner point if it satisfied the conditions $d_{min}^{2}\leq {\left|℘-{℘}^{+}\right|}^{2}\leq d_{max}^{2},\;d_{min}^{2}\leq {\left|℘-{℘}^{-}\right|}^{2}\leq d_{max}^{2}$ and *ϕ*≤*ϕ*_*max*_, where ℘ is the point, ℘^+^ is the *k*^*th*^ clockwise neighboring point of ℘ and ℘^−^ is the *k*^*th*^ anti-clockwise neighboring point of ℘. If |*a*| = *a* = |℘−℘^+^| is the distance between ℘ and ℘^+^, |*b*| = *b* = |℘−℘^−^| is the distance between ℘ and ℘^−^, |*c*| = *c* = |℘^+^−℘^−^| is the distance between ℘^+^ and ℘^−^. Thus, the opening angle of the triangle, named as *ϕ* can be determined by using law of cosine: $\phi ={cos}^{-1}(\frac{a^{2}+b^{2}+c^{2}}{2ab})$. Subsequently, for each point ℘, there may exist zero, one or more than one *ϕ*-values. So that the minimum value of *ϕ* among all *ϕ* -values is picked out as *ϕ*—value of the point ℘.

**Fig 8:**
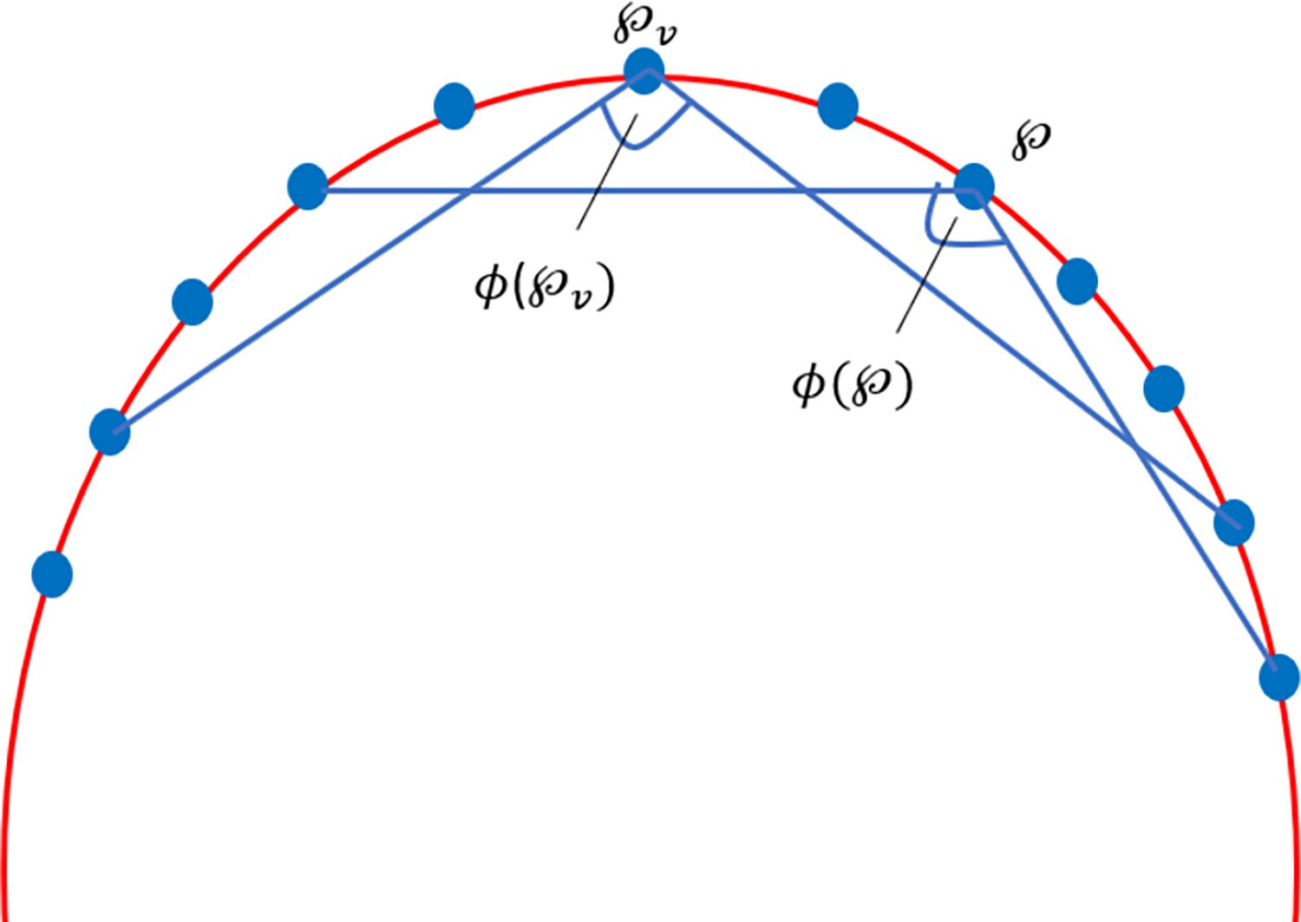
Corner point detection demonstration–Phase 2.

Phase 2: In this phase, some additional points are annihilated. A potential corner point ℘ from the initial phase is cast away if it possesses a sharper valid neighboring point ℘_*v*_: *ϕ*(℘)>*ϕ*(℘_*v*_). Here, the candidate point ℘_*v*_ is a valid neighboring point of ℘ if ${\left|℘-{{℘}_{v}}^{-}\right|}^{2}\leq d_{max}^{2}$ or if it is next to ℘. Visually the phase 2 is showcase in Fig 8.

**Fig 9:**
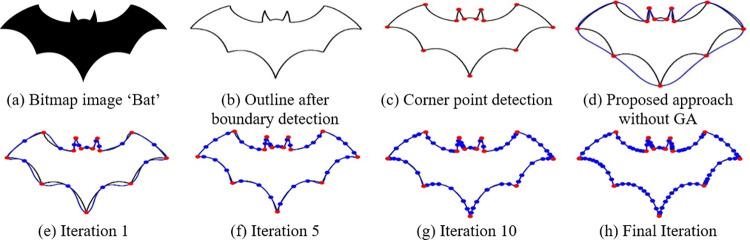
Some iterations of GA using proposed algorithm for the bit map image ‘Bat’.

**Fig 10:**
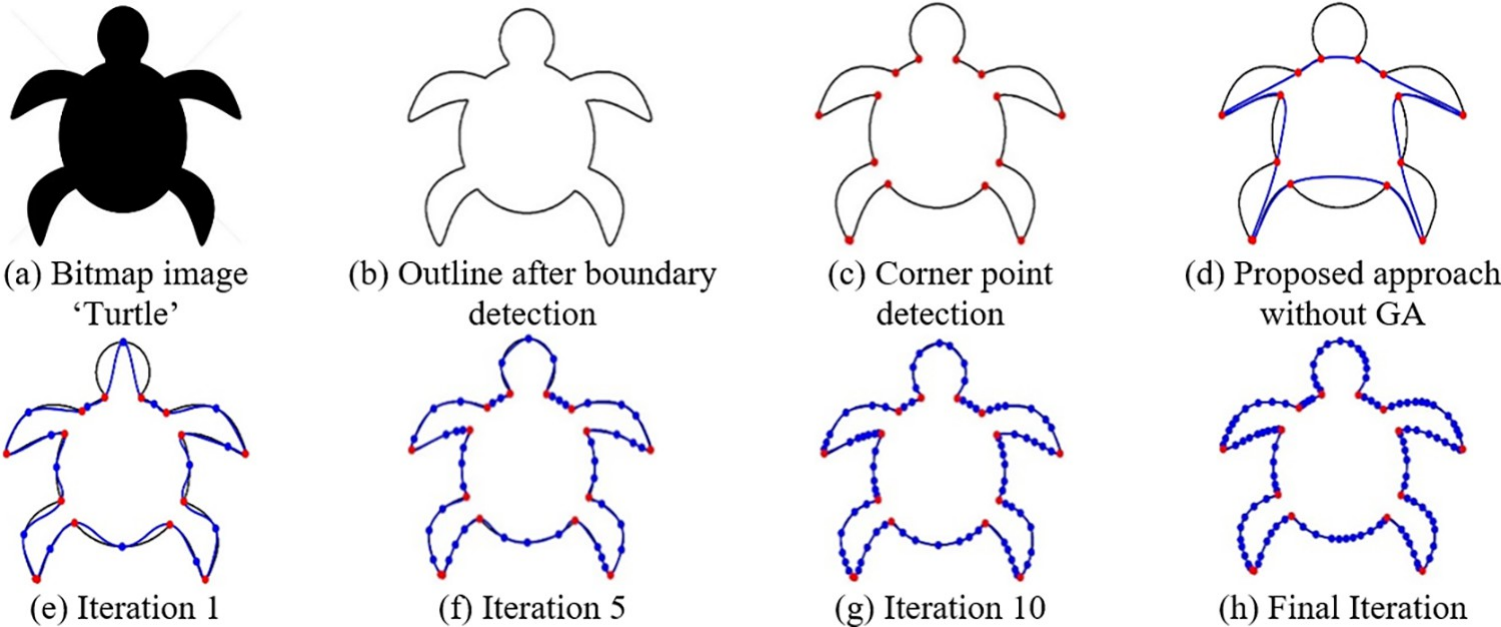
Some iterations of GA using proposed algorithm for the bit map image ‘Turtle’.

**Fig 11:**
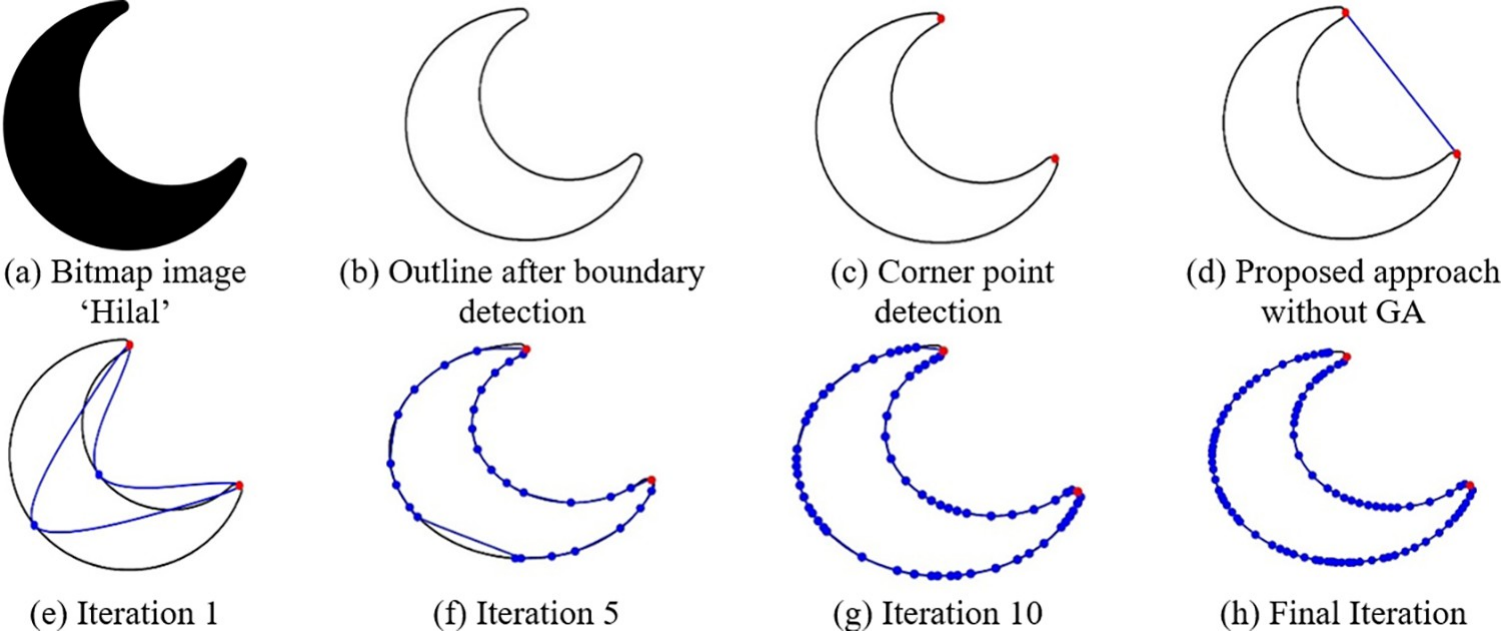
Some iterations of GA using proposed algorithm for the bit map image ‘Hilal’.

Additionally, the corners are of paramount importance as they separate the data into distinct segments. Following the segmentation, the data values can be made up as *P*_*i*,*j*_ = (*x*_*i*,*j*_, *y*_*i*,*j*_); *i* = 1,2,3,…,*n* and *j* = 1,2,3,….,*m*_*i*_ with *n* denoting the number of segments and *m*_*i*_ representing the number of data points in the *ith* segment. Here, *P*_*i*,*j*_ signifies the *jth* point within the *ith* segment. Visually the new detected corners of the boundaries shown in Figs 9(B), 10(B) and 11(B) can be seen in Figs 9(C), 10(C) and 11(C), respectively. Table 2 provides information on the number of contour points and the count of detected corner points for sample images.

**Table 2:** Particulars of contours and corner points together with intermediate points for different iterations along with time elapse.

Image Name	# of contours	# of data points	# of initial corner points	# of intermediate points				Time Elapse
				1^st^	5^th^	10^th^	Final	(sec)
Bat	1	1225	13	11	28	47	88	2.1841
Turtle	1	1806	14	13	31	58	109	2.8693
Hilal	1	1667	2	2	17	41	98	2.1942

### 5.3 Problem description

The objective is to generate a curve that closely approximates the original one (the contour), essentially finding suitable parameter values that yield the desired curve outline. The proposed approach effectively transforms the initial continuous problem into a discrete optimization problem. In this formulation, each shape parameter corresponds to an individual gene within a chromosome’s bit string, as shown in Fig 5. In this representation, when a gene is set to 1, assign a value to the corresponding shape parameter, and when it’s set to 0, do not assign a value. If the shape parameters are expected to fall within the interval [a, b], they are determined within this range (a, b). So that the initial population consists of K individuals, each having a gene length of L. These genes are randomly initialized with 0s and 1s. It is vital to notice that the corner points are chosen prior to establishing the first population. The goal of this strategy is to keep these essential locations since they play an important function in forming the contours of the desired forms.

In order to find the value of parameter, the fitness function denoted as *S*_*i*_ will calculate as the squared sums of the distances between *P*_*i*,*j*_ ‘s and their corresponding parametric points on the curve as follows:

$$S_{i}=\sum_{j=1}^{m_{i}}{\left[P_{i}^{*}(\zeta_{j})-P_{i,j}\right]}^{2}\;i=0,1,\ldots .,n-1$$

To achieve the best fit of the curve to the given data, such values for the parameters *ω*_*i*_ are essential that minimize the sums *S*_*i*_. GA is employed to optimize these values for the fitted curve. A random population for *ω*_*i*_ is considered to initialize this optimization process. Therefore, iteratively applying optimization operations to this collection leads us to the optimal values of *ω*_*i*_.

### 5.4 Curve fitting

The identification of corner points initiates the division of the image boundary into smaller segments. Spline Interpolation outlined in Section 2 has been applied to estimate each segment of the boundary. The spline involves a parameter *ω*_*i*_, in its description. Initially, the values of *ω*_*i*_ chosen randomly. Once an initial estimation for the segment is derived, GA is executed to determine the optimal values of *ω*_*i*_. It plays a decisive role in obtaining improved approximations leading to optimal solutions.

### 5.5 Breaking segment

In certain instances, the iterative refinement process may not yield a sufficiently satisfactory best fit for specific segments. In such situations, divide the segment into even smaller ones at locations when the gap between the boundary and the parametric curve surpasses a pre-established threshold. These designated points are referred to as intermediate points. For each of these new segments, a fresh parametric curve is fitted, as illustrated in Figs 9(E)–9(H), 10(E)–10(H) and 11(E)–11(H). Table 2 presents the number of intermediate points acquired during the optimization of the QTB-Spline for various iterations of the Genetic Algorithm.

The gap between the boundary and the parametric curve is frequently represented as the squared distance between each point of a digitalized curve and its corresponding point on a parametric curve [17, 18]. However, this work uses the technique described in [19] to calculate the distance between the board and the parametric curve. This approach defined the distance (d) between a point *P*_*i*,*j*_ on the digitized curve and its corresponding parametric point P*(*ζ*_*i*,*j*_) on the curve as the largest difference between the abscissas or ordinates of under discussion points.

### 5.6 Algorithm

The entire process of computing the desired manipulation of the outline, the optimal QTB-spline curve, can be condensed into the following algorithm.

1. **Input:** Provide the raster image for vectorization.
2. **Boundary Detection:** Extract the boundary from the image provided in **Step 1.**
3. **Corner Identification:** Detect corners points from the boundary using the method described in Section 5.2.
4. **Curve Fitting:** Fit curves to the detected corners using the proposed QTB-Spline.
5. **Optimization:** Use GA to get the best optimal value of the parameters *ω*_*i*_.
6. **Termination:** If the curve, achieved in **Step 4**, is optimal then STOP and Go TO **Step 7**, otherwise, locate appropriate intermediate points as described in Section 5.5, and RETURN to **Step 4**.
7. **Output:** Generate the vectorized representation of the image.

## 6. Experimental results

The curve fitting approach presented in Section 5 has been put into practice on various images. Fig 9(A) displays the original image (b) showcases the image’s outline, (c) exhibits the corner points for bit map image ‘Bat’, and (d)—(h) reveal the fitted outlines without and with GA, (e) represents the first iteration and (h) shows the final iteration, instant of this, (f) and (g) shows the experimental results of fifth and tenth iteration, with a threshold of 3, utilizing Genetic Algorithms in conjunction with both corner and intermediate points. A similar description can be applied to Figs 10 and 11 for bit map images ‘Turtle’ and ‘Hilal’ respectively. The time required to apply this proposed method to different images is detailed in Table 2.

**Fig 12:**
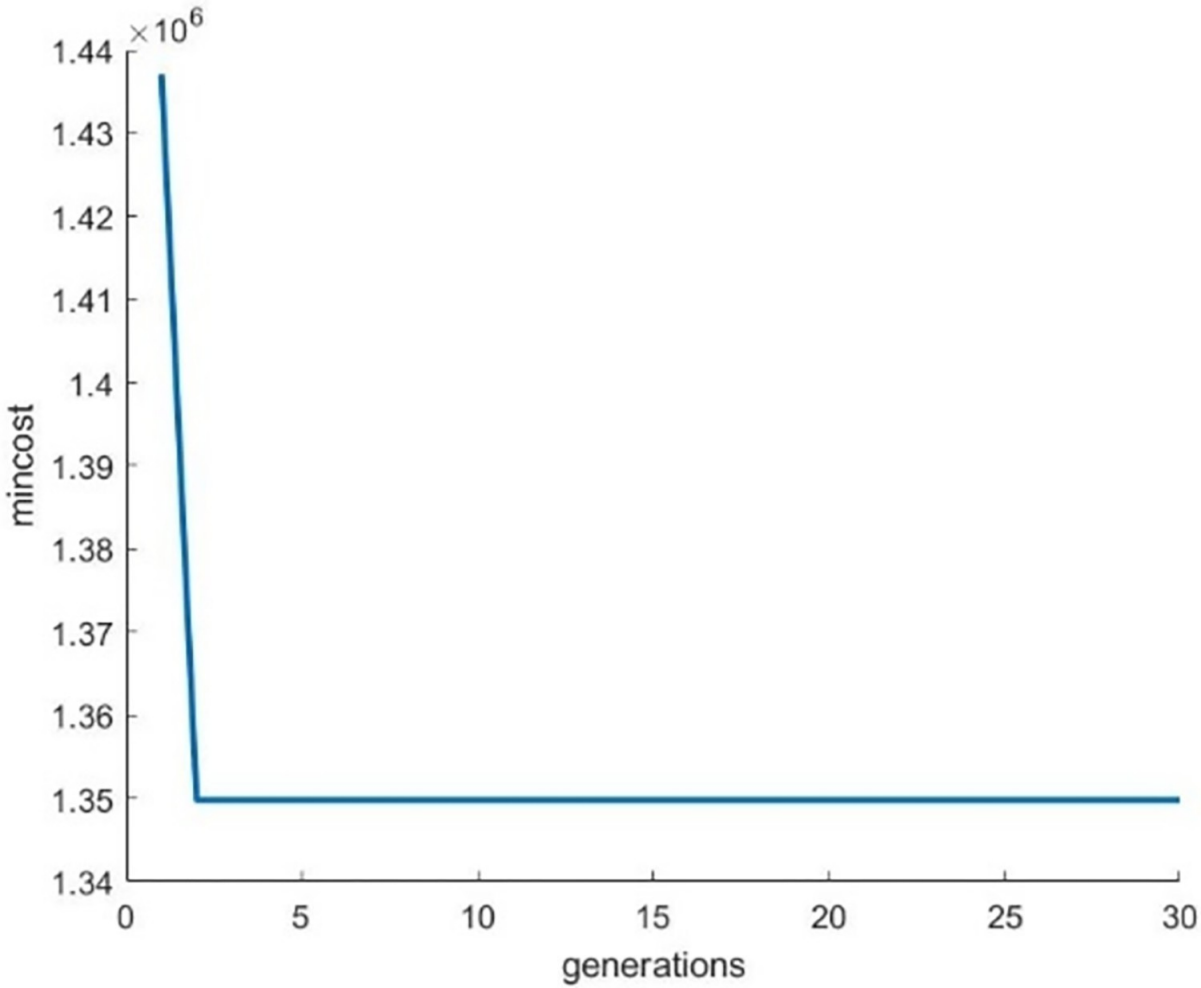
Behaviour of the fitness function for the image “Bat”.

**Fig 13:**
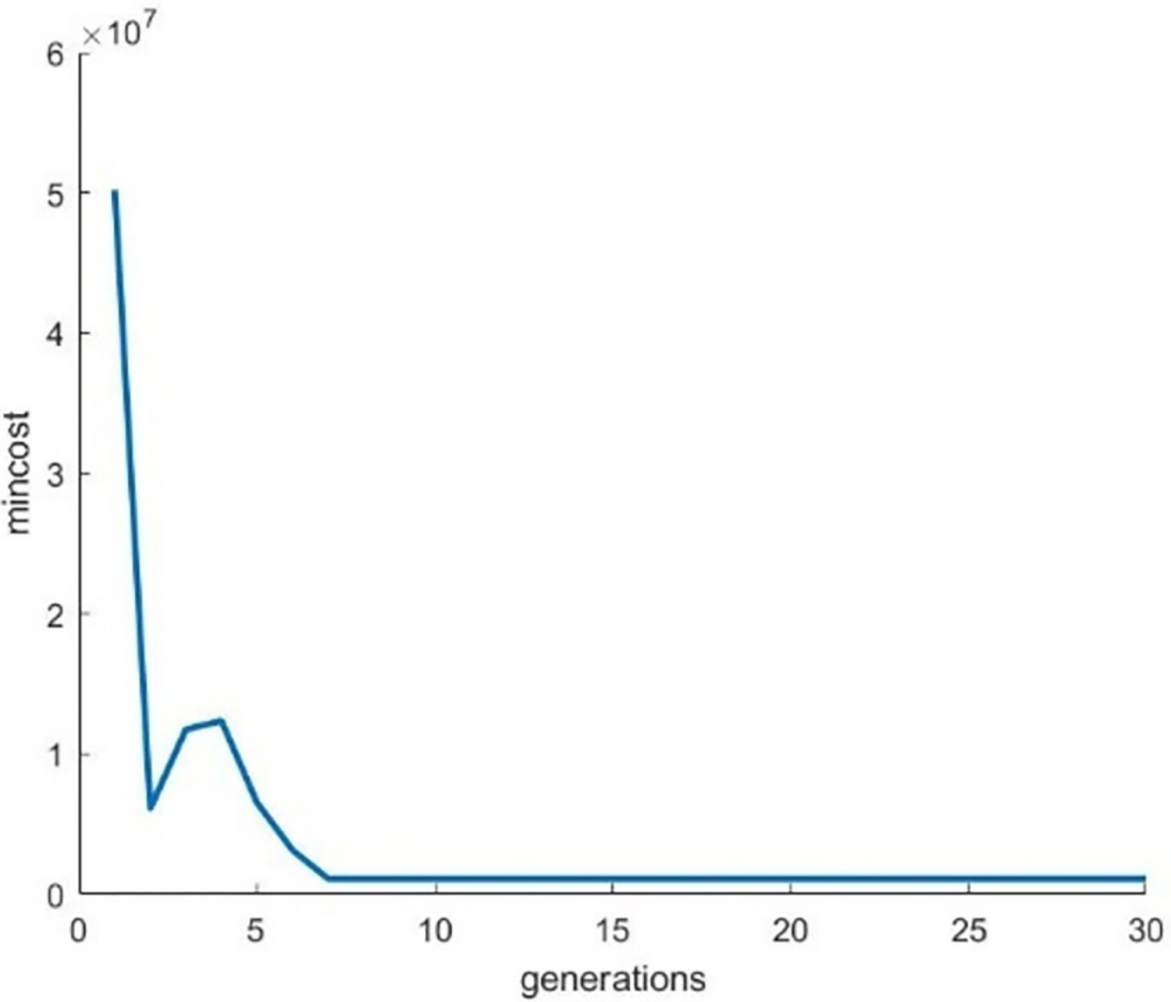
Behaviour of the fitness function for the image “Turtle”.

**Fig 14:**
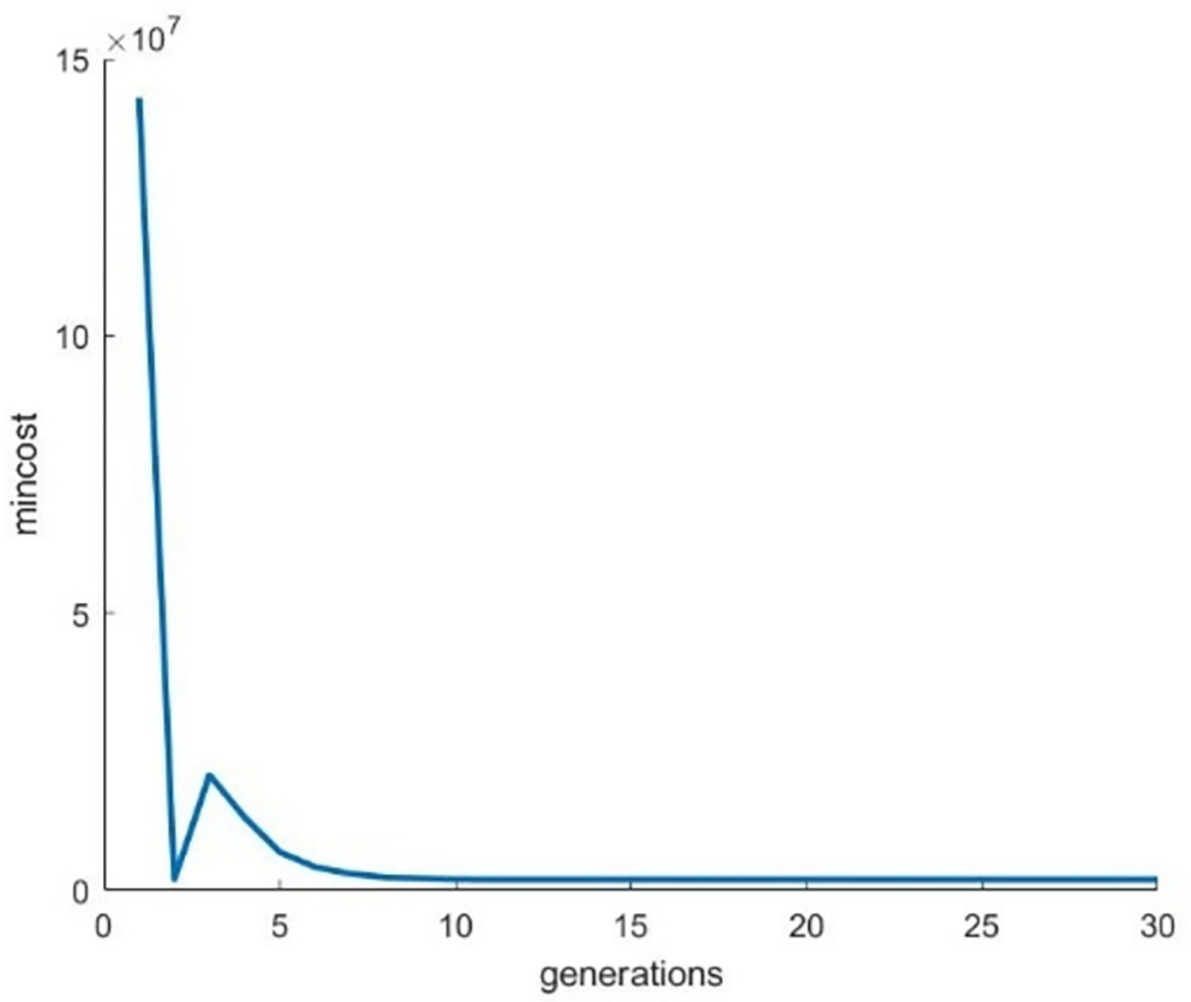
Behaviour of the fitness function for the image “Hilal”.

Figs 12–14 illustrates the behavior of the fitness function when running the Genetic Algorithm repeatedly on images of Bat, Turtle and Hilal respectively. From these graphical visuals, it is evident that the lowest value of the cost function is attained after the 30th iteration.

## 7. Comparative & subsequent studies

The proposed scheme introduces several noteworthy advantages over its predecessors. Firstly, it exhibits enhanced computational efficiency compared to the methods outlined in references [3, 9–12], particularly evident in its ability to swiftly process vectorized outlines of images for planar objects with extensive datasets. Quantitative comparisons of computational time, as depicted in Tables 2 and 3, underscore this efficiency when placed side by side with existing techniques. In future the proposed method could extend to handle three-dimensional vectorization of shapes. The method would involve adapting the existing algorithm to incorporate depth information, enabling accurate representation of volumetric outlines in addition to planar contours.

**Table 3:** Quantitative comparison of total time taken in seconds together with datapoints.

Algorithm	Data Points	Time elapsed (Sec)
Scheme Presented in [3]	1225	2.4840
	1806	3.8741
	1667	2.3019
Scheme Presented in [9]	1641	164.17
	1250	100.58
	693	70.297
Scheme Presented in [10]	673	6.075
	1005	6.7
	975	9.395
Scheme Presented in [11]	1641	749.511
	1250	286.033
	693	486.722
Scheme Presented in [12]	1641	37.0251
	1250	28.5504
	693	12.2587

## 8. Conclusions

A method is proposed to optimize the vectorization of raster image outlines. This technique employs the Genetic Algorithm to fine-tune a trigonometric spline to match the digital outline of the images. By initiating the search process from well-identified starting points (initially detected corner points), it achieves improved convergence results. The overall process encompasses several phases, including image outline extraction, corner point detection within the outlines, curve fitting, and the potential addition of extra knot points as necessary. The concept of the genetic algorithm is harnessed to optimize the shape parameters within the B-Spline description. This method ultimately yields optimal outcomes for approximating the vectorization of digital contours derived from generic shapes. It offers an optimal alignment while maintaining computational efficiency in the curve-fitting process. Importantly, the proposed algorithm operates autonomously and do not require human intervention.
